# A multi-site single-blind clinical study to compare the effects of STAIR Narrative Therapy to treatment as usual among women with PTSD in public sector mental health settings: study protocol for a randomized controlled trial

**DOI:** 10.1186/1745-6215-15-197

**Published:** 2014-05-29

**Authors:** Marylene Cloitre, Clare Henn-Haase, Judith L Herman, Christie Jackson, Nadine Kaslow, Constance Klein, Michaela Mendelsohn, Eva Petkova

**Affiliations:** 1 VA Palo Alto Health Care System, 795 Willow Road, Menlo Park, CA 94025, USA; 2 New York University Langone Medical Center, 550 First Ave, New York, NY 10016, USA; 3 Harvard University Medical School, Cambridge Health Alliance, 1493 Cambridge, St. Cambridge, MA 02139, USA; 4 VA New York Harbor Healthcare System – Manhattan Campus, 423 E 23rd Street, New York, NY 10010, USA; 5 Grady Department of Psychiatry, Health System, Emory University School of Medicine, 1648 Pierce Drive, NE, Atlanta, GA, 30322, USA; 6 The Nathan S. Kline Institute for Psychiatric Research, of New York State Office of Mental Health (OMH), 140 Old Orangeburg Rd, Orangeburg, NY 10962, USA

**Keywords:** PTSD, Community mental health clinics, Women’s health, Flexible service delivery

## Abstract

**Background:**

This article provides a description of the rationale, design, and methods of a multisite clinical trial which evaluates the potential benefits of an evidence-based psychosocial treatment, STAIR Narrative Therapy, among women with posttraumatic stress disorder (PTSD) related to interpersonal violence who are seeking services in public sector community mental health clinics. This is the first large multisite trial of an evidence-based treatment for PTSD provided in the context of community settings that are dedicated to the treatment of poverty-level patient populations.

**Methods:**

The study is enrolling 352 participants in a minimum of 4 community clinics. Participants are randomized into either STAIR Narrative Therapy or Treatment As Usual (TAU). Primary outcomes are PTSD, emotion management and interpersonal problems. The study will allow a flexible application of the protocol determined by patient need and preferences. Secondary analyses will assess the relationship of outcomes to different patterns of treatment implementation for different levels of baseline symptom severity.

**Discussion:**

The article discusses the rationale and study issues related to the use of a flexible delivery of a protocol treatment and of the selection of treatment as it is actually practiced in the community as the comparator.

**Trial registration:**

Clinicaltrials.gov identifier: NCT01488539.

## Background

Public sector mental health services are the disproportionate recipients of patients with trauma histories and, not surprisingly, such settings report high rates of post-traumatic stress disorder (PTSD) in their patient population. Exposure to traumatic stressors is reported by 62% to 98% of public sector patient samples and prevalence rates of PTSD range from 19% to 43% [1-3]. PTSD tends to occur more frequently in women than in men, and is most commonly related to multiple instances of interpersonal violence including those reaching back to childhood experiences of sexual and physical abuse [1]. PTSD related to interpersonal violence is particularly pernicious as it is associated with significant psychiatric co-morbidity, high rates of suicide attempts, abuse of alcohol and drugs, and chronic interpersonal and relationship difficulties [4]. At least 40 single-site randomized trials of cognitive behavioral therapy for PTSD have been conducted [5,6], and these trials have resulted in the identification of several efficacious therapies. However, to date, the evaluation of the effectiveness of these therapies in large community settings has been limited to military populations and military clinics.

Given the pervasive presence of trauma histories and PTSD in patients seeking treatment in public sector mental health settings, there is great need to evaluate evidence-based treatments in these settings and to disseminate those shown to be effective. In addition, research is needed on processes that facilitate the adoption and effective use of EBTs in community settings.

This article describes a National Institute of Mental Health (NIMH) funded study in which the effectiveness of an evidence-based treatment for women with PTSD related to interpersonal violence will be assessed and compared to treatment as usual in low-income community public sector outpatient clinics. The intervention to be evaluated is STAIR Narrative Therapy, a two-module, sequential treatment that was specifically developed to treat women with chronic PTSD, common co-morbid symptoms, and substantially impaired functioning [7,8]. The first module emphasizes skills training in affective and interpersonal regulation (STAIR) and has the primary goal of improving daily life functioning, while the second module (Narrative Therapy) incorporates and focuses on the review and re-appraisal of trauma memories [9].

The proposed study utilizes a hybrid efficacy/effectiveness design [10]. This type of trial focuses on the evaluation of the benefit of a treatment when applied in a community service setting under research sampling and assessment conditions. Its design has features that maintain internal and construct validity, while the treatment is evaluated under ‘real world’ clinical conditions that enhance external validity and generalizability. Generalizability features include engagement of sites with ethnically and racially diverse populations, few inclusion and exclusion participant criteria, use of community clinicians, a flexible approach to treatment application, and clinical training and supervision consistent with service environments. Aspects of efficacy designs that are maintained in the proposed study are: random assignment of patients into STAIR Narrative Therapy (SNT) or Treatment as Usual (TAU), use of reliable and standardized diagnosis assessment measures, and use of a manual to guide the protocol treatment and relatively intensive quality assurance of diagnostic assessments and of adherence to the treatment protocol.

The study also incorporates several elements of implementation science research. Clinicians are encouraged to use the SNT protocol in a flexible manner within clear limits, according to the symptoms, needs, and preferences of the specific patient. We view the incorporation of clinician-driven decisions regarding application of the protocol as an important ingredient in facilitating the use and dissemination of EBTs in the community. We will conduct exploratory analyses identifying implementation patterns and their relationship to outcomes for patients and also examine the influence of therapist and organizational characteristics likely to impact implementation. These include, but are not limited to, therapists’ attitudes about the intervention and administrative support for implementation of the intervention within the service. The study also introduces web-based technology as a resource intended to strengthen clinical networks and maintain use of study materials about which we will obtain qualitative data from clinicians regarding its perceived value.

This article provides a description of the study design and reasons for design decisions such the selection of TAU as a comparator and the use of flexible delivery of SNT. These choices were intended to increase clinical realism and the generalizability of findings. However, they also added to the complexity of the study. The selection of TAU, which in a multisite study differs from site to site, requires consideration about how to characterize and assess TAU interventions and analytic techniques that take between-site variability in TAU into account. Similarly, the use of a flexible delivery of the test treatment incorporates clinician judgment and supports patient-centered care, but requires careful consideration about how to define and ensure adherence to the protocol. The study description below provides an example of the issues encountered in hybrid efficacy-effectiveness clinical trials. We present the rationale for our decisions and the scientific literature that guided them.

The study has two aims: (1) to evaluate the effectiveness of SNT relative to TAU primarily in regards to PTSD symptom reduction and secondarily in regards to treatment attendance, attrition, and therapist and patient satisfaction, and (2) to explore the relationship between variations in SNT treatment implementation and outcome relative to baseline severity of illness (for example, PTSD severity, GAF score). In addition, the study will obtain qualitative data concerning therapist and staff attitudes about contextual variables (for example, administrative support, clinic resources) including the use of technology (SNT resource website) that facilitate or impede implementation of the EBT and will explore their relationship to intervention delivery and outcome. These data will be collected via interviews conducted at the midpoint and endpoint of the clinical trial.

## Methods

### Design

**Figure 1:**
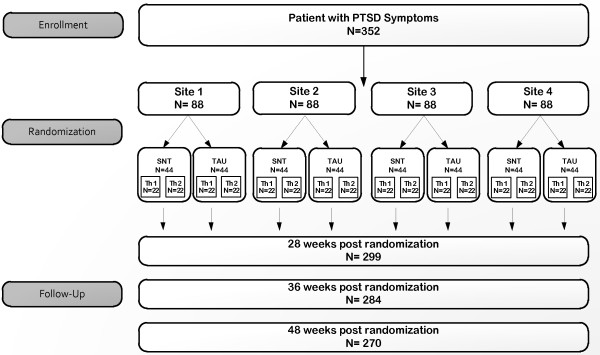
Diagram of participant flow.

The study design is a randomized controlled trial of treatment in US public sector mental health outpatient settings (see Figure 1). A minimum of four sites will participate in the study. Within each site, participants will be randomized into either SNT or TAU and each treatment condition will be implemented by a minimum of two therapists. Each therapist delivers only one of the interventions. Selection of sites includes demonstration of ability to recruit a minimum of three study participants per month based on number of new trauma patients entering the clinic on a yearly basis and on the percent of such patients with PTSD diagnoses. Other features required of sites include the presence of Institutional Review Board (IRB) and administrative resources and infrastructure to provide fiscal management, reporting, and quality assurance required by the funder, NIMH.

### Participants

Participants must be women aged between 18 and 65 years. They must have a primary diagnosis of PTSD according to DSM-IV criteria [11] with a Clinician Administered PTSD Scale (CAPS) score no lower than 40 [12]. The PTSD symptoms must be a result of interpersonal violence and participants must have at least one clear trauma memory.

Exclusion criteria are as follows: substance dependence and severe substance abuse disorders, current psychotic symptoms, unmedicated mania or bipolar disorder; prominent current suicidal or homicidal ideation (a plan or intent *versus* a wish) or a suicide attempt within the past 3 months; self-injurious behaviors in the last 3 months requiring medical attention; cognitive impairment indicated by chart diagnoses or observable cognitive difficulties; and current involvement in a violent relationship defined as more than casual contact (for example, dating or living with an abusive partner). Individuals for whom a co-morbid disorder is identified as of greater clinical significance than PTSD as defined by the SCID (for example, severe eating disorder, severe Borderline Personality Disorder) are for clinical and ethical reasons referred to services that treat these disorders. Severe BPD is defined as having at least the following three symptoms in concert: (1) fear of abandonment, (2) unstable self, and (3) frequent and ongoing self-injurious or suicidal behavior under stress. Thus, inclusion/exclusion criteria allow individuals with substantial psychiatric difficulties including: severe depression, moderate substance dependence, eating disorders, moderate borderline personality disorder, and history of psychotic disorders or currently medicated psychotic disorders.

### Assessment

Consistent with the plans for flexible application of SNT and with the variable length of naturally occurring TAU, treatment duration is free to vary but assessments will be conducted at fixed intervals [13]. This will allow a direct comparison of the two treatment conditions, SNT and TAU, regarding improvement and differential rates of change over time. Participants will be informed that the assessments will be conducted on a fixed schedule, independent of the number of sessions or expected duration of the therapy. After randomization, assessments will be conducted according to the following schedule: during the 28th week, the 36th week, and the 48th week from randomization, after which the treatment trial will end for participants in both conditions and referral to another therapist or clinic will be provided as needed.

The measures and domains are summarized in Table 1. The primary outcome is PTSD symptom severity as measured by the CAPS [14] and remission from the disorder, defined as no longer meeting the diagnosis of PTSD and having a CAPS score no greater than 20 [12]. Secondary outcomes are emotion regulation self-efficacy as measured by the Difficulties in Emotion Regulation Scales (DERS; [15]), interpersonal problems as measured by the Inventory of Interpersonal Problems (IIP; [16]), and global functioning as measured by the Global Assessment of Functioning (GAF) from the SCID [17].

**Table 1:** Assessment schedule by domain for study hypothesis

**Measure**	**Domain**	**Enrollment**	**Baseline**	**Sessions 1-16**	**Mid-treatment week 12**	**28-week assessment**	**36-week assessment**	**48-week assessment**
Informed consent		X						
Screen		X						
Primary outcome								
CAPS	PTSD Dx & Symptom Severity		X			X	X	X
Secondary outcomes								
IIP	Interpersonal problems		X		X	X	X	X
DERS	Emotion regulation		X		X	X	X	X
GAF	Functional status		X			X	X	X
Baseline psychopathology								
SCID I	Axis I disorders		X			X	X	X
SCID-II Screen	Axis II disorders		X			X		
BSI	General distress		X		X	X	X	X
SF-36	Subjective health functioning		X			X	X	X
HSUF	Recent and ongoing health service utilization		X			X	X	X
Treatment and treatment process								
PCL-5	PTSD self-report		X	Alternate sessions	X	X	X	X
TSI-Diss	Dissociation self-report		X	Alternate sessions	X	X		
WAI	Therapeutic alliance				Sessions 1 to 5	X		
TES	Satisfaction with treatment and environment					X		

BSI: Brief Symptom Inventory, CAPS: Clinician Administered PTSD Scale, DERS: Difficulties in Emotion Regulation Scales, GAF: Global Assessment of Functioning, HSUF: Health Service Utilization Form, IIP: Inventory of Interpersonal Problems, PCL: 5 PTSD Checklist, SCID: Structured Clinical Interview for Diagnosis, TES: Treatment Experience Scale, TSI: Trauma Symptom Inventory, WAI: Working Alliance Inventory.

Several additional assessment measures are obtained at baseline to evaluate overall mental and physical health. These include the Brief Symptom Inventory (BSI) [18] and the Trauma Symptom Inventory dissociation subscale (TSI) [19], the Structured Clinical Interview for Diagnosis (SCID) for all Axis I disorders [17], and the SCID II screen to identify probable Axis II disorders [20]. Current health status is measured by the SF-36 [21] and health service utilization by the Health Service Utilization Form (HSUF) (Berman M, Franklin M, Cox J, Foa E, Miller S: *The Quality Of Life Self-Report Instrument,* unpublished). Additional secondary measures focusing on more complex aspects of PTSD included at the major assessment points for exploratory purposes are the Complex Trauma Symptoms Questionnaire (CTSQ) [Mendelsohn M, Herman JL, Cloitre M, Henne-Hasse C, Jackson C, Kaslow NJ, Lanius R: *The Complex Trauma Symptom Questionnaire*, Unpublished manuscript], the Affective Experience Questionnaire (AEQ) [Frewen P: *The Affective Experience Questionnaire*, Unpublished manuscript], the Self Compassion Scale (SCS) [22], the Relationship Scales Questionnaire (RSQ) [23], the Connor-Davidson Resilience Scale (CDRS) [24], The Traumatic Dissociation Scale (TDS) [25], and the Somatoform Dissociation Questionnaire (SDQ) [26].

At the beginning of every therapy session, a five-item empirically validated suicide checklist, the Suicide Behavior Monitoring Form (SBMF) [27], is completed by the therapist. At the end of every other session (2, 4, 6, 8, and so on) study participants complete a brief (5-minute) assessment of PTSD symptoms (PCL-5) and the TSI dissociation subscale to track change in symptoms over the course of treatment. Treatment process is assessed using the Working Alliance Inventory-12 item (WAI-12) [28]. Satisfaction with the treatment and its perceived usefulness as well as satisfaction with the clinical environment (warmth, organization) is assessed using a measure developed for this study and is completed at the end of every treatment by participants as well as therapists for each treatment case.

### Enrollment

Enrollment involves a two-stage process. Potential candidates are self-referred or referred by a clinician in the service. The project coordinator at each site completes an informed consent process with the potential candidate and a face-to-face screen to determine whether or not the potential participant is willing to adhere to study conditions, is likely to have PTSD and to meet other inclusion and exclusion criteria. If the basic criteria appear to be satisfied, the project coordinator schedules a second appointment to complete the baseline assessment to confirm the diagnosis of PTSD and all other study criteria. Any necessary contact with existing providers occurs after the assessment to ensure that the project coordinator has a complete mental health history, to make certain that the other provider(s) are aware of the potential participant’s interest in enrolling in the study, and to ensure their understanding of the study protocol. The candidates are contacted within 1 to 5 days of the baseline assessment regarding their eligibility for the study. Patients will receive payment for completing baseline, 28-, 36-, and 48-week assessments to cover costs of travel and other expenses (for example, childcare).

### Randomization

Eligible participants are randomly assigned to either SNT or TAU. The study manager located at the coordinating site provides each clinical site with the treatment assignment upon a phone or text request by the project coordinator. Participants are randomized to STAIR Narrative Therapy or TAU in blocks of random size (2, 4, or 6) stratified by site. The randomization lists were generated by ‘R’, an open source statistical program. The treatment condition to which the participant is randomized is not revealed until she arrives at her first treatment session. Thus attrition that occurs between acceptance for treatment and the first session cannot be attributed to treatment assignment. The interval between completion of baseline assessment and first treatment session is expected to be no more than 2 weeks. If circumstances occur such that attendance at first session occurs more than 4 weeks after completion of assessment, the CAPS is re-administered. The outcome assessors are located at the coordinating site and are blind to the participants’ treatment condition. Unblinding concerning treatment allocation is permissible if a study participant experiences a serious adverse event and reporting to the Data Monitoring Committee (DMC) and the funder (NIMH) is required.

### Treatment

#### ***STAIR Narrative Therapy (SNT)***

SNT is a 16 session manualized treatment that is delivered in 60-minute weekly sessions in an individual format. The STAIR module focuses on resolving problems in daily living through skills training in emotion management and interpersonal functioning. The first four sessions focus on increasing emotion regulation skills including awareness of feelings, self-soothing exercises, and distress tolerance. The second four sessions focus on development of interpersonal skills and include emotion management in the context of social/interpersonal interactions, exploration and revision of maladaptive interpersonal schemas, effective assertiveness, and increased flexibility in interpersonal expectations and behaviors depending on context. Narrative Therapy is a modified version of prolonged exposure. Two interventions have been added to standard prolonged exposure approaches: (1) ‘grounding’ exercises immediately after exposure, and (2) cognitive reappraisal of the meaning of the trauma, with a focus on interpersonal schemas. Expectations about relationships derived from the trauma narrative are identified and validated, and then contrasted with alternative beliefs or ‘working models’, developed during STAIR that may be more adaptive for current relationships. Repeated practice of interpersonal skills between sessions functions as a skills-building intervention as well as a form of *in-vivo* exposure regarding generalized fears concerning social interactions and relational engagement [7].

SNT is flexibly applied following the guidelines established in a previous study that evaluated the effects of flexible delivery of the treatment [29]. The study found outcomes to be equivalent to those obtained in a previous trial that required strict adherence to the protocol [7]. In the current study, treatment can range from 16 to 24 sessions. The type and number of sessions are determined by the clinician, whose decisions are guided by the needs of the individual patient. The study protocol allows (a) skipping protocol sessions, (b) repeating sessions, and (c) having non-protocol sessions. There are constraints, such that each of the three components of the treatment has a minimum number of sessions as follows: (1) three sessions of skills training in emotion regulation; (2) four sessions of skills training in interpersonal effectiveness; (3) five sessions of narrative work; and (4) two sessions that bookend the treatment, namely the introduction to treatment and termination sessions. This formula requires that a total of 14 sessions are committed to a particular set of interventions. The clinician can adhere very closely to the minimum requirements and use only two additional sessions to deviate from the protocol (for a total of 16) or can deviate rather substantially with the addition of 10 sessions (for a total of 24). Additional sessions may include more emotion regulation, interpersonal, narrative, or non-protocol sessions, as determined by the clinician.

### The selection of TAU as a comparator

The comparator in this study is TAU as it is delivered in each of the community sites. Substantial consideration went into making this decision. Some clinical trials use a standardized TAU and develop manuals of supportive counseling or generic problem-centered therapy for implementation [30,31], while others have a TAU condition in which the treatment implemented is that which is typically provided in the setting [32,33]. The benefit of standardizing TAU is that it creates a control condition that is well defined and constant across sites. This reduces the heterogeneity of the outcomes from the TAU condition, thus increasing the power of the tests for the differences between conditions (for a given sample size), and internal validity. However, external validity is limited. That is, the comparison does not allow an assessment of a question of clinical interest and practical import, which is whether SNT is more effective than current practice. Standardized TAU is not necessarily like the treatments used by community providers, but rather is a constructed treatment approximating what therapists actually do in practice. Accordingly, if the results indicated that SNT was more effective than a standardized TAU, the findings might be dismissed by community organizations as having little relevance to their practice. The weakness of a design that uses TAU as it naturally occurs in a clinic, particularly in a multisite study, is that there is likely to be wide variation in TAU from site to site, raising the concern that between-site variability will reduce the power of the study to detect clinically meaningful differences between the treatments. In addition, the nature of TAU often remains unidentified, as content is rarely monitored.

We decided to use TAU as it naturally occurs in clinics, in order to be able to determine the ‘real world’ value of SNT and the study sample size was selected to account for the extra variance due to TAU heterogeneity. Meta-analyses of multisite studies indicate that the variability in outcomes between sites tends to be smaller than the within-site differences between the treatments [34]. We identified the range of effect sizes that have been found for naturally occurring TAU, based on those available in the few PTSD and the more numerous PTSD/SUD studies that have used community TAU as a comparator. This range was used to guide our power calculations described later in this paper (see Statistical Analysis section). In addition, we developed a manual, itemizing interventions from a range of other treatment modalities (psychodynamic, cognitive behavioral, supportive, mindfulness/meditation), that would be used to evaluate the content of TAU. These data will help to characterize TAU at each site, and will also allow some analyses about interventions within TAU that are associated with good outcome.

### Additional treatment

Participants may stay on medication, attend self-help groups, and receive treatment for mental health problems other than PTSD. The presence of concurrent psychotherapy is unacceptable only if the treatment is a PTSD treatment or a trauma-focused intervention. Participants who develop problems requiring additional inpatient or outpatient treatment may receive additional treatment as determined by the therapist, the site participating investigator, and the supervisor of the treatment condition to which the participant has been assigned. The amount and type of additional treatment is monitored and documented across all sites using a standardized form (that is, Health Utilization Form (HSUF)). After completing the study treatment, participants may seek additional treatment outside the study as clinically indicated and this is similarly monitored with the HSUF in the follow-up assessments at 36 and 48 weeks.

### Discontinuation

Treatment is discontinued for participants who become actively suicidal or homicidal, engage in an uncontrolled episode of alcohol or drug abuse that requires immediate treatment, or requires inpatient psychiatric treatment. Participants may also be discontinued from the study if they resume or initiate a relationship in which they are being physically or sexually abused. Treatment can also be discontinued when further study treatment is determined by the treating clinician and supervisor to be clinically inappropriate.

Treatment is also discontinued when participants fail to attend three consecutive therapy sessions or a total of six sessions throughout the course of the treatment, without a reason judged by their therapist or site team to be acceptable. For intent-to-treat (ITT) purposes, all participants, including those who are terminated early, are assessed at 28 weeks, 36 weeks, and 48 weeks after randomization. Participants who are discontinued from the study for any reason are referred to more appropriate types of treatment as indicated.

### Therapist selection, training, and supervision

Potential therapists for the study are identified by the site PI in conjunction with the study PI. Therapists are required to have a master’s or doctoral degree in clinical or counseling psychology, social work or psychiatric nursing, or a medical degree with specialization in psychiatry. Therapists at each site are assigned to either SNT or TAU. Therapists assigned to TAU are provided with the option of obtaining training in SNT at the end of a 2-year commitment as a TAU therapist. It is expected that most therapists would be women. However, male therapists may participate based on clinic culture and staffing patterns. We will explore the potential relationship between degree, gender, years of experience, and theoretical orientation to implementation patterns and outcome.

Training in SNT includes reading the workbook [9], which describes the theory and principles of treatment, observing a videotape of a ‘gold standard’ 16-session treatment that follows the study SNT treatment manual, and completing a training case. The training case requires completing all eight sessions of skills training and at least four sessions of exposure. The case sessions are audiotaped and supervised session by session. The test case is rated for adherence by two trained adherence raters who report deviation from protocol to the supervisor. The SNT adherence manual rates adherence to core interventions as a categorical assessment (yes or no). Successful completion of a training case is defined as achieving a 75% adherence rating on a minimum of 12 sessions which include sessions from the emotion regulation, interpersonal skills, and narrative modules. If the therapist does not reach this level of adherence, the therapist completes additional mock intervention sessions with the supervisor until she meets the criteria of 75% adherence. If adherence is less than 50%, the therapist will complete a second case. SNT supervision occurs weekly at each site with both the site PI and one of the SNT trainers (Drs. Cloitre and Jackson).

### Fidelity monitoring

SNT and TAU treatment sessions are audiotaped and sent to the coordinating site for coding. SNT sessions are coded for therapist adherence. TAU sessions are coded to characterize their elements and to assess for presence of specific interventions in SNT using an adapted version of an established coding procedure, the Hamburg Psychotherapy Process Scale (HaPPS-O) [35]. Coding of key therapeutic elements is completed for psychodynamic, cognitive behavioral and supportive therapy, mindfulness/meditation practices as well as the specific core elements of SNT. Coding of SNT and TAU will allow a comparison of the degree to which elements in TAU may overlap with those of SNT.

SNT tapes are reviewed on a weekly basis by 10 PhD students with a Masters degree in psychology and a minimum of 2 years of clinical experience with public sector patients. Training on adherence rating requires a half-day review of the adherence rating protocols followed by practice ratings of eight tapes (five SNT and three TAU). For the SNT condition, two out of three sessions (or 67% of cases) are rated. For the TAU condition, a total of three tapes per case is randomly sampled (excluding first and last session). Each tape is assessed by two raters. The PI (MC) reviews 10% of each rater’s tapes to guard against rater drift. The PI also meets quarterly (or more frequently if needed) with raters to maintain consistent quality of ratings and to resolve identified discrepancies.

### Use of technology to enhance resources and support sustainability

There is accumulating evidence that web-based technology is an efficient and effective method of supporting clinical training [36]. We will incorporate a web-based platform to (1) make standard clinical materials readily available and easily downloadable during the treatment trial itself and (2) provide a platform through which treatment materials can be managed and clinical networks across the sites can be maintained and expanded after the study is completed. Reactions to the use of the web, including perceptions of its impact on sense of professional support, self-efficacy, and work satisfaction, as well as its potential as a tool for enhancing dissemination of evidence-based treatment will be obtained through two sets of focus group interviews with clinicians; one will be conducted around the half-way mark of the trial and the other at the end of the trial.

### Data management

The data management site will provide paper case report forms (CRFs) for the collection of all data required by the study. Data will be collected at the study site on paper and entered by the project coordinator on a daily basis to computerized user-friendly electronic version of the forms with a backend process that will identify missing, illogical, out of range, and inconsistent values for each data element. The data will be reviewed for completeness, timeliness, and accuracy on a nightly basis. If any errors are detected, an automatic electronic message will flag the error for correction. Personnel from the Data Management site will be available for consultation if needed to facilitate the correction. When no further errors are detected, the data management site will ‘lock’ the study data from further modification. No personal identifying information is included in the dataset. Original data files with identifying information are double-locked (that is, kept in locked cabinets and locked rooms) at each of the clinical sites.

### Statistical analysis

#### Sample size determination

The sample size was determined based on the primary hypotheses regarding superiority of SNT compared to TAU immediately post-treatment (at week 28 post-randomization) and with respect to course of symptoms during follow up (from 28 to 36 and 48 weeks post-randomization). Reviews of the literature indicated that the pre-post effects with STN were consistently large with Cohen’s d effect size greater than 0.80 [7,8,29]. In contrast, a review of the relatively small PTSD literature that has used TAU as a comparator and the somewhat larger PTSD/SUD literature revealed that effect sizes associated with TAU ranged widely from 0.3 to 1.2 [32,33]. The sample size was determined to allow 80% power of a two-sided test with level of significance α = 0.05 to detect (a) differences immediately post treatment with respect mean CAPS of size Cohen’s d = 0.35; and (b) differences in the course of symptoms during follow up that result in a difference of size Cohen’s d = 0.8 at 6-month follow-up. The necessary sample size depends on the variation in the TAU effects between sites, the clustering due to therapist (which reduced the effective sample size by a magnitude that depended on the correlation between outcomes of participants treated by the same therapist), the clustering due to site, (which reduces the variance of the difference between treatments, akin to pairwise *t*-test being more efficient than 2-sample *t*-test) and the correlation between the three repeated observations on a patients ranging from. We project 20% dropout prior to the first treatment session and overall 30% missing post-randomization data. Considering ranges of possible values of these factors and selecting most conservative likely combination of them determined that a total n = 352 patients from a minimum of four sites would be necessary. This sample size also allows sufficient power to detect difference of 20% in the remission rates at 28 weeks and an increase of this difference during follow-up by 25%. Regarding the hypotheses concerning the secondary outcomes, the study provided sufficient power to detect effects similar to those for symptoms severity measured by CAPS as the secondary measures are continuous.

### Hypotheses testing regarding SNT as compared to TAU

The primary hypothesis of the study is that SNT will be more effective than TAU for the treatment of PTSD, as well as for frequently co-occurring problems in emotion regulation, relationships, and general functioning. All analyses will be on the intent-to-treatment (ITT) sample with imputation for missing data. The intent-to-treat sample is defined as all randomized participants who attend at least the first therapy session at which they are notified about their treatment assignment. Testing of all hypotheses (including those concerning differences at a particular time point), will be based on models for longitudinal data [37]. Continuous outcomes such as severity of PTSD symptoms, emotion regulation problems, and interpersonal problems will be modeled with linear mixed effects model (MEM), while dichotomous outcomes, such as PTSD remission, will be modeled using generalized linear mixed effects model (GMEM).

Given the small number of sites, the ability to estimate the site variability with sufficient precision is limited. For this reason, the site and site-by-treatment and site-by-treatment-by-time interaction effects will be modeled as fixed effects and the variation between sites will be examined informally. The main effect of site will be included in all models whether or not it is significant. In order to protect against erroneous omission of important site effects, the statistical significance of the interactions involving site and treatment will be judged at level α = 0.15, and the site-specific treatment effects will be estimated with 95% CIs, rather than considered nil when not reaching statistical significance at α = 0.05 level.

### Exploratory analyses regarding SNT patterns of implementation

One aim of the study highly relevant to the concerns of implementation research, *per se*, is our plan to assess the potential benefits and/or drawbacks of allowing flexible delivery of the protocol. Flexible implementation variables will follow those used in Levitt et al. [29] and will include: number of sessions (ranging from 16 to 24), different types of flexible adaptations (repetitions, skips of key interventions) for the treatment in its entirety, as well as the number of sessions and occurrence of each type of adaptation within each intervention component (emotion regulation skills, interpersonal skills, and narrative work).

Each participant in the SNT condition will have values for these variables defining how the treatment was implemented for her. We plan to identify patterns of implementation associated with patient outcomes for patients at different levels of baseline severity. We will categorize patients according to level of baseline severity defined in various ways (PTSD severity, GAF score, number of co-morbidities). For each of these categorization schemes, patterns of implementation associated with outcome will be identified: excellent outcome (those achieving remission), good outcome (improvement but not remission), and poor outcome (no change or worsening). In addition to traditional regression models, recursive partitioning methods [38,39] will be utilized, allowing the identification of implementation patterns (groupings of implementation variables) as they are associated with baseline severity and outcome, and leading to the identification of optimal implementation patterns for given baseline characteristics.

### Qualitative interviews regarding contexual characteristics

We will introduce and explore the influence of contextual variables including clinician characteristics (for example, age, gender, professional training, years of experience) and organizational characteristics obtained from clinician and patient perception of organizational support and organizational resources (support staff, material resources) for both SNT and TAU at the end of the treatment. All participants will assess their treatment experience at the end of their treatment and, in parallel, the clinicians will evaluate their perception of organizational support and resources for each particular patient. In addition, focus groups for clinicians and for study administrative staff will be conducted twice during the life of the study, once at the midpoint of the study’s treatment trial (when approximately half of the participants have completed the treatment) and the second near the end of the treatment trial.

## Discussion

This study is designed to evaluate the implementation of an evidence-based treatment for PTSD in public sector community settings with community clinicians. To date, there have been no large-scale clinical trials of an evidence-based PTSD treatment in the general community. While PTSD clinical trials have been completed in the VA service system, its specific veteran population as well as the policies, structures, and processes that guide implementation efforts in the VA may not generalize to public sector community mental health settings. This study, with its large sample and multi-site design is expected to yield evidence regarding the effectiveness of SNT relative to usual care on PTSD and range of comorbidities, as well as secondary outcomes such as health status and health service utilization in a chronically traumatized and impoverished patient population.

The study will also contribute to knowledge in implementation science. Specifically, the study will test a flexible implementation strategy of the study treatment, in which the therapist and the patient pair will select a subset of interventions within the three core intervention modules (emotion management, interpersonal/social skills, and narrative therapy) based on the patients’ needs and preferences. The study therefore emphasizes a patient-centered approach to the delivery of an evidence-based treatment and may be the first to provide data regarding the outcomes of treatment as they relate to specific patterns of intervention use. The sample size, along with appropriate use of state-of-the-art statistical techniques, will allow us to identify patterns of implementation (variations in selected interventions) associated with both good and bad outcomes, and in particular, as they relate to different levels of severity in psychiatric illness.

The study will more broadly contribute to implementation science in that both qualitative and quantitative data will be obtained from several different types of stakeholders in the public sector setting including patients, clinicians, and administrators concerning factors that impeded and facilitated the implementation process. This information will contribute to the evidence base seeking to identify factors and strategies that support and facilitate change in community mental health systems. The delivery of psychotherapy in poverty level settings has special challenges. In such settings, experiences of chronic and repeated violence, unemployment, limited material resources (for example, funds for transportation, rent, and childcare), and variable access to healthcare create a backdrop of chronic stress and unpredictability that adversely affects the ability of the mental health system to serve patients effectively. While these circumstances are highly challenging, they are unfortunately quite common in public sector settings. This study is important in that it will provide information about the process and outcomes associated with introducing innovations into the context of a highly stressed type of mental health system.

## Trial status

Currently screening and recruiting for participants.

## Abbreviations

AEQ: Affective Experience Questionnaire; BSI: Brief Symptom Inventory; CAPS: Clinician Administered PTSD Scale; CDRS: Connor-Davidson Resilience Scale; CTSQ: Complex Trauma Symptoms Questionnaire; DERS: Difficulties in Emotion Regulation Scales; EBT: Evidence Based Treatment; GAF: Global Assessment of Functioning; GMEM: Generalized Linear Mixed Effects Model; HaPPS-O: Hamburg Psychotherapy Process Scale; HSUF: Health Service Utilization Form; IIP: Inventory of Interpersonal Problems; ITT: intent-to-treatment; MEM: Mixed Effects Model; NIMH: National Institute of Mental Health; PCL: PTSD Checklist; PTSD: Post Traumatic Stress Disorder; RSQ: Relationship Scales Questionnaire; SBMF: Suicide Behavior Monitoring Form; SCID: Structured Clinical Interview for Diagnosis; SCS: Self Compassion Scale; SDQ: Somatoform Dissociation Questionnaire; SNT: STAIR Narrative Therapy; STAIR: Skills Training in Affective and Interpersonal Regulation; TAU: Treatment As Usual; TDS: Traumatic Dissociation Scale; TSI: Trauma Symptom Inventory; WAI-12: Working Alliance Inventory-12 item.

## Competing interests

The authors declare that they have no competing interests.

## Authors’ contributions

MC conceived of the study aims and design and completed the first draft of the manuscript. CH-H participated in the conception and design of study and manuscript revision. JLH participated in the conception and design of study and data collection and manuscript revision. CJ participated in the conception and design of study, quality assurance of treatment, and manuscript revision. NK participated in the conception and design of study, data collection, and revision of the manuscript. CK developed the data management and integrity procedures and participated in the manuscript revision. MM participated in the conception and design of study, data collection, and revision of the manuscript. EP developed the data analytic plan, and participated in the conception and design of study and the revision of the manuscript. All authors read and approved the final manuscript.

## Authors’ information

Marylene Cloitre is the Associate Director of Research at the National Center for PTSD Division of Dissemination and Training, VA Palo Alto Health Care System. She is also Research Professor in Psychiatry and in Child and Adolescent Psychiatry at the NYU Langone Medical College in New York City. Her area of clinical research is the development, testing, and dissemination of psychosocial therapies for PTSD.

## Study roles and responsibilities

There are six sites in the study: a coordinating site, a data management site, and four clinical sites. The coordinating site has responsibility for all aspects of the study and is led by MC. The data management site led by CK has responsibility for the development of data collection and data monitoring strategies and guidelines. The biostatistician (EP) is responsible for the data analytic plan and supervision of the data analyses. CJ is responsible with MC for the supervision of the therapists in the test treatment. The clinical sites (CH-H, JLH, NK, MM) are responsible for the recruitment and treatment of the PTSD patients. All study sites are required to obtain IRB approval at their site as relevant to their role in the study. The coordinating site IRB is an ‘umbrella’ document and cannot be approved until all of the sites submit their approved IRBs. Recruitment of participants cannot be initiated until all sites, including the coordinating site, has approved IRB. Informed consents have been developed at each clinical site and include information about the study, and policies regarding protection of confidentiality and compensation for those who suffer harm from the trial. Modifications to the protocol and informed consents are reviewed and approved at each individual site and at the coordinating site. Modifications are reported to the DMC on a bi-annual basis and on a quarterly basis to the funder.

All study staff at all sites receive training in confidentiality protection of participant data and in the recognition and procedures associated with adverse events and serious adverse events. This information is documented in the study Protocol and Procedures manual. This manual provides information about all aspects of the study and is updated by the coordinating site as needed (for example, for clarification of or change in procedures). All sites keep an updated copy of the Protocol and Procedures manual. There are twice-monthly administrative meetings for site PIs and coordinators which include the review of changes and/or questions about study protocol and procedures.

A Publication and Data Access Policy document identifies who will have access to the final trial dataset, authorship guidelines, and plans for communication of results by investigators and sponsor.

The DMC comprises four individuals who have expertise in the implementation of multisite trials (2), in the treatment of PTSD (2), and in statistical analyses for multisite trials (1). One individual is expert in both treatment of PTSD and implementation of multisite studies. The DMC is independent from the sponsor and the members do not have any competing interests. The DMC charter contains description of plans for managing adverse events/serious adverse events, plans for interim analyses, and stopping guidelines.

The sponsor of the study is NIMH and the contact person is Ben Vitiello (bvitiell@mail.nih.gov).
